# Medical genetics needs assessment: An online cross-sectional survey from Nepal

**DOI:** 10.1016/j.gimo.2025.103477

**Published:** 2025-11-24

**Authors:** Pratiksha Gyawali, Binaya Shrestha, Kelly Beharry, Gareema Agarwal, Shane C. Quinonez

**Affiliations:** 1 Department of Clinical Biochemistry, Kathmandu University School of Medical Sciences, Kavrepalanchok, Nepal; 2 Department of Pathology, Kathmandu University School of Medical Sciences, B.P Highway, Kavrepalanchok, Nepal; 3 University of Michigan Medical School, Ann Arbor, MI; 4 Division of Pediatric Genetics, Metabolism and Genomic Medicine, Department Pediatrics, University of Michigan, Ann Arbor, MI

**Keywords:** Capacity building, Genetic disorders, Genetic services, Needs assessment, Nepal

## Abstract

**Purpose:**

Medical genetic services remain limited in low- and middle- income countries, such as Nepal, leading to poor health outcomes for individuals affected by genetic disorders. This study aimed to assess perspective and characterize current practices and attitudes toward genetic services among health care providers in Nepal.

**Methods:**

A web-based survey was completed by 131 clinicians across multiple disciplines, exploring participant demographics, experience with genetic services, and perceived barriers to genetic testing and counseling.

**Results:**

Although 42% of respondents reported regularly caring for patients with suspected genetic disorders, 77% of providers reported difficulties with obtaining genetic testing. The most frequently cited barriers included limited laboratory availability (28%), cost (26%), and logistical challenges (19%). Many respondents reported confidence in discussing disease recurrence risk (63%), treating genetic disorders (40%), and providing genetic counseling (48%), and the majority (86%) expressed interest in furthering their genetic education because only 19% felt their current genetics knowledge was sufficient.

**Conclusion:**

This study highlights a clear demand for accessible, affordable, in-country genetic services in Nepal and underscores the need for investment in clinical training and capacity building to improve access and outcomes for patients with genetic disorders.

## Introduction

Genetic disorders and congenital anomalies (CAs), defined as structural, functional, and metabolic malformations present at birth, are important public health problems given their significant impact on children worldwide.^1^ Although genetic disorders result from a chromosomal and/or single gene defect, CAs results from the complex interplay between genetic factors and environmental influences, such as maternal nutrition, infection, and drug exposure.^2^ Globally, approximately 240,000 newborns die within 28 days of birth annually as a result of these CAs, with an additional 170,000 children dying between 1 month and 5 years of age.^3^ Children who survive go on to face higher chances of physical, cognitive, and social disabilities throughout their lives, which place significant financial, emotional, psychological, and social burdens on their families and account for a staggering 25.3 to 38.8 million disability-adjusted life-years worldwide.^3^^,^^4^

Although the impact of genetic disorders and CAs affect individuals worldwide, their impact is felt most by low- and middle-income countries (LMICs), where 94% of anomalies occur annually.^3^ The reasons for this are multifactorial, but low income has been identified as an indirect determinant of CAs, in addition to higher fertility rates, limited prenatal cell-free DNA screening, and limited options for termination of pregnancies affected by a suspected genetic disorders—all which are present in LMICs.^3^^,^^5^ Unfortunately, nearly all low-income countries and many middle-income countries lack the necessary personnel, capital, technology, infrastructure, and public and medical education capabilities needed to implement genetic services to address this burden of genetic disorders and CAs.^6^ For genetic disorders specifically, trained personal and diagnostic services are extremely limited in almost all LMICs.^7^ GeneTests shows that, of the 603 international laboratories offering genetic testing, none are located in low-income countries, and only 20 are found in middle-income countries (www.genetests.org).^8^ Addressing this gap in services has been difficult for many LMICs; however, a number of them have utilized the principles of Community Genetics to introduce medical genetics as a way to address genetic disorders and CAs.^9^ Community Genetics aims to prevent CAs and genetic disorders at the population level while simultaneously providing genetic services to affected individuals and their families.^9^ Community genetics has been defined as “the art and science of the responsible and realistic application of health and disease-related genetics and genomics knowledge and technologies in human populations (communities) to the benefit of individual persons”.^10^ Unfortunately, given the diverse number of issues affecting LMICs, medical genetic services for the prevention and management of CAs have received little attention in most LMICs to date. A Health Needs Assessment is an inclusive, evidence-based approach that uses epidemiological data to provide information for planning, introducing and improving health care programs, and has been suggested as a useful initial step when considering the introduction of a new service to a region or country.^6^

Nepal is a landlocked country in South Asia with limited medical genetic services and a recently identified CA prevalence of 5.8 per 1000 live birth.^11^ Currently, there are a few medical geneticists who were trained in Srilanka and pioneered the establishment of first medical genetics unit in the country.^12^ Therefore, we aimed to understand the current status of medical genetic services and utilization of family history throughout Nepal by conducting an online Health Needs Assessment of Nepalese physicians. We focused on providers’ education, experience, and preferences regarding medical genetic services and identified key areas that require attention to increase the availability of these services in Nepal. The data collected as part of this study will provide necessary information for the future expansion of genetic services in the country.

## Materials and Methods

### Survey design

This is a quantitative observational cross-sectional online survey conducted among Nepalese health professionals. We conducted the survey between March 2023 to December 2023. The study conduct and reporting comply with the Strengthening the Reporting of Observational Studies in Epidemiology guidelines for cross-sectional studies.^13^

### Survey sample and administration

The survey was conducted using the web-based survey tool Qualtrics with questions developed by the research team and adapted from the previously validated survey used for similar purposes in Ethiopia and Ghana.^14^ A total of 40 items were included in the survey. The survey collected no personal identifying information and was pretested to assess the readability and comprehension. The first part of the survey covers participants’ general demographics and professional information. The second part addresses questions related to genetic services, genetic testing, and genetic counseling, whereas the third part focuses on the utilization of family health history in patient care. Participants were instructed to not consider COVID-19 testing as genetic testing for the purposes of this survey.

Participation was voluntary with respondents providing informed consent before completing the survey; no incentive was offered. Convenience sampling was utilized to survey health professionals working in various practice settings in Nepal, including government and private academic institutes, clinics, hospitals, and laboratories. The network of health care professionals included mostly board-certified clinicians, academicians, consultants, nurses, researchers, and public health and laboratory professionals. The survey link was distributed electronically within investigator P.G.’s network using communication platforms such as Email, WhatsApp, Facebook messenger, and Viber. The invitation was sent through Viber to the Medical School Alumni and other association group with the remainder of potential participants sent individual invitations by P.G. All advertisements used the same recruitment materials and information, which described the survey as a “medical genetics needs assessment in Nepal.”

Health professionals in the process of obtaining board certification, interns, and students were not included in the survey. The survey was made available throughout the duration of the study. A total of 423 health professionals were sent the survey.

### Survey setting

Nepal is a landlocked country in South Asia situated mainly in the Himalayas though with 3 major geographic regions: Himalayan (mountainous), hilly, and the Tarai (plain). There is limited genetic services in the country with less than 10 medical geneticists in the country and no certified genetic counselors. The health care system in Nepal functions on a mixed public-private model with public hospital able to provide subsidized services. The Nepal National Health Insurance Program started in 2016 and is a voluntary program covering care at established public and private facilities. Genetic services do not typically fall under this program given a large number of genetic testing require testing outside of Nepal though limited work has been done assessing practices and coverage patterns throughout the country.

### Survey data analysis

Data from uncompleted surveys were not used and considered as withdrawn, with only completed surveys analyzed. Data were extracted from Qualtrics and descriptive statistics were computed using Microsoft Excel. Continuous and categorical data are reported as frequency and percentage with 95% confidence interval.

## Results

### Demographics

A total of 131 out of 423 health professionals completed the survey, producing a response rate of 31%. The demographics of participants are shown in Table 1. Of the participants, 52.7% identified as female with 47.3% identifying as male. Most participants (57%) were between 30 and 39 years of age. All 7 Nepali Provinces were represented in the sample, though 60.3% were from Province 3 (Bagmati Province), the second most populated province and home to the capital city of Kathmandu. Province 1, the most populated Nepali province, had the second highest representation in the sample with 13.7% of participants. The majority of participants (82.4%) reported having some degree of postgraduate education, with a wide spectrum of professional designations. Residents made up 34.4% of participants, whereas Assistant Professors accounted for 22.9%. Most respondents (59.5%) were from academic institutions. The most common specialty of participants was Pathology (33.8%), Internal Medicine (19.2%), and Surgical Specialties (15.4%). Other specialties included, but were not limited to, Physiology, Dermatology, Pediatrics, Anatomical Sciences, and Dentistry.

**Table 1:** Characteristics of the study participants

Characteristics	Number (Percent)
Age Distribution	
Under 30	36 (28.1%)
30-39	73 (57.0%)
40-49	19 (14.8%)
Gender Distribution	
Female	69 (52.7%)
Male	62 (47.3%)
Province Distribution	
Province 1	18 (13.7%)
Province 2	17 (13.0%)
Province 3	79 (60.3%)
Province 4	5 (3.8%)
Province 5	8 (6.1%)
Province 6	2 (1.5%)
Province 7	2 (1.5%)
Postgraduate Education Participation	
No	23 (17.6%)
Yes	108 (82.4%)
Designation Summary	
Other	9 (6.7%)
Resident	45 (34.4%)
Assistant Professor	30 (22.9%)
Associate Professor	20 (15.3%)
Consultant	15 (11.5%)
Lecturer	12 (9.2%)
Hospital/Institution Summary	
Academic Institution	78 (59.5%)
Non-Academic Institution	53 (40.5%)
Specialty Summary	
Pathology	44 (33.8%)
Internal Medicine	25 (19.2%)
Surgical Specialties	20 (15.4%)
Other	41 (31.6%)

### Genetic services experience and practices

**Figure 1:**
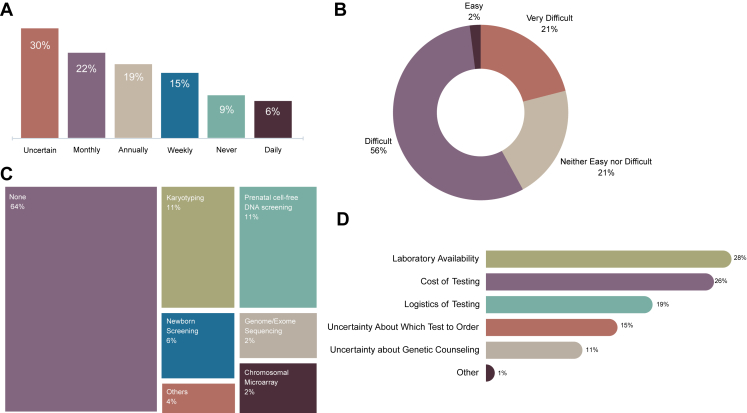
**Information regarding participants’ practices related to medical genetics services.** A. Frequency of encountering patients with confirmed or suspected genetic disorders. B. Perceived challenges in genetic testing for Nepalese patients. C. Reported availability of genetic testing. D. Major barriers to genetic testing.

Sixty-three percent of all participants reported never ordering genetic testing, whereas 37% reported ordering genetic testing in the past. Fifty-three percent of participants were aware of a genetic testing laboratory, compared with 47% who were not. Figure 1 shows information regarding participants’ practices related to medical genetics services. Figure 1A shows 42% of respondents see a patient with a confirmed or suspected genetic disorders at least monthly, with 6% of participants seeing such patients daily. Interestingly, 30% of participants were uncertain of the frequency with which they see a genetics patient. As shown in (Figure 1B), 77% of participants reported performing genetic testing on Nepalese patients was either difficult or very difficult, with only 2% reporting the testing was easy to have done in Nepal. Sixty-four percent of respondents reported no genetic testing is performed at their home institution, despite a wide array of reported tests being conducted there (Figure 1C). When considering difficulties with ordering genetic testing, laboratory availability was the most cited barrier (28%), followed by cost of testing (26%) and testing logistics (19%) (Figure 1D). Uncertainty regarding test selection and genetic counseling were also highlighted as frequent barriers.

**Figure 2:**
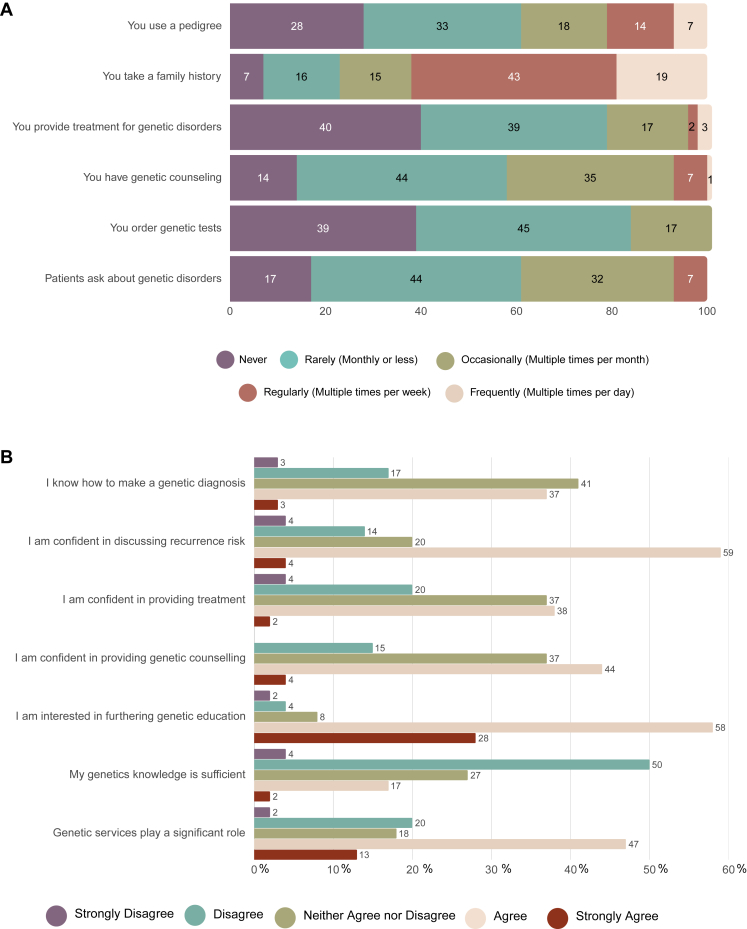
**Responses focused on participants’ medical genetics practices and self-assessed knowledge.** A. Clinical practice related to medical genetics. B. Genetic knowledge and confidence in genetic diagnosis.

Figure 2 shows responses focused on participants’ medical genetics practices (Figure 2A) and self-assessed knowledge (Figure 2B) regarding genetic services. Participants reported collecting a family history much more frequently than using a pedigree in clinical practice, with 19% frequently collecting a family history during patient encounters, compared with 7% of respondents who frequently (multiple times per day) use a pedigree. Eighty-four percent of respondents reported never or rarely ordering genetic testing, whereas 61% of respondents reported never or rarely encountering patients inquiring about genetic disorders. Most respondents (79%) reported either never or rarely (monthly or less) providing treatment for genetic conditions, and 58% of participants either never or rarely provide genetic counseling.

Regarding genetic knowledge, 40% of participants reported agreeing or strongly agreeing that they knew how to make a genetic diagnosis, whereas 41% neither agreed nor disagreed with the statement. Most respondents (63%) felt confident discussing recurrence risk, 40% felt confident providing treatment for genetic disorders, and almost half of respondents (48%) felt confident providing genetic counseling. The majority of participants (86%) expressed interest in furthering their genetic education because only 19% feeling their genetics knowledge was sufficient.

Comparing the 3 most common specialties (Pathology, Internal Medicine, and Surgical Specialties) we noted differences and similarities between many of the questions asked. Specifically, we noted that all 3 specialty respondents described their ability to get genetic testing completed as either “Neither easy nor difficult,” “Difficult,” or “Very Difficult.” For items assessing agreement or disagreement with various statements response options ranged from Strongly Disagree (score of 1) to Strongly Agree (score of 5). For the statement “I frequently obtain a family history when caring for patients” Pathologists averaged a score of 3.65, Surgical Specialties averaged 4.27, and Internal Medicine averaged 4.45. Internal Medicine providers were also noted to be the most confident in their ability to collect a family history (4.15) compared with Pathologists (3.83) and Surgeons (3.93). All 3 specialists had similar scores for the item assessing confidence in their ability to provide genetic counseling based on a family history (Pathologists 3.22, Internal Medicine 3.25, and Surgical Specialties 3.13). All 3 specialties also similarly felt their genetics knowledge was not sufficient for their clinical practice with all scores < 3.0, and all were interested in furthering their genetic medicine education with scores > 4 with all respondents in Pathology and Internal Medicine Strongly Agreeing with this item.

In addition to the survey responses, we also allowed participants to add any additional comments regarding how to provide better care for Nepali patients with confirmed or suspected genetic disorders. The most common additional comments related to the need for in-country genetic testing infrastructure that offers affordable genetic testing for Nepali patients. Additional frequent comments highlighted the need for better training of Nepali providers in medical genetics and genetic counseling.

## Discussion

Here, we report the results from medical genetics needs assessment among health care providers from Nepal. The information obtained from this work will provide necessary information regarding the needs and focus of future efforts aimed at expanding medical genetics in the country. In general, we identified that Nepali providers are seeing patients with confirmed or suspected genetic disorders but feel limited in their abilities to provide treatment and services for these patients. This gap in services is multifactorial and relates not only to a need for more education, but also a lack of affordable in-country diagnostics and genetic counseling services.

In our survey, only 37% of participants reported sending genetic testing in the past, although 53% of respondents were aware of a genetic testing laboratory. There is incredibly limited genetic testing available in Nepal, with the majority of testing sent out of country to India or Europe. This limited testing capability was reflected in the item related to respondents’ ability to get genetic testing performed on patients, in which 77% of participants reported that it was either difficult or very difficult. Not surprisingly, laboratory availability and the cost of testing were cited as the most common barriers to genetic testing. It is important to note that testing sent out of the country requires patients to pay for testing out of pocket, which is often not feasible for most patients.^8^ This has been identified as a common limitation to performing genetic testing in other LMICs as well.^9^

In Nepal, an in-country lab would also increase the likelihood of test access for all patients, given that patients with certain medical conditions, such as cancer and sickle cell disease, receive a governmental subsidy for in-country services related to those diagnoses. Although the use of international laboratories facilitates access to genetic testing, the associated cost remains a substantial barrier. In addition, many physicians report having limited knowledge in selecting appropriate tests and must independently coordinate the complex logistics involved in sample shipment. Even with in-country testing, as was reported in Ethiopia, clinicians often lack the necessary genetics education to make recommendations for testing, with this often falling to the laboratory director.^15^ This suggests a synergistic role between the medical genetics’ education of providers and the development of in-country genetic molecular diagnostics, whereby the lack of one negatively affects the ability of the other to function fully.

Interestingly, 36% of respondents reported that their home institution offered some degree of genetic testing, including prenatal cell-free DNA screening and chromosomal microarray, although only a few responses reported these services. As mentioned, we are unaware of these technologies being available in Nepal, and it is possible this relates to a lack of provider education or to institutions that advertise these services but ultimately send samples out of the country to a different lab. Further work will be needed to better investigate the full breadth of testing being offered in Nepal.

Review of provider practices show providers regularly participating in a number of medical genetic services, such as delivering genetic counseling and using a pedigree. Importantly, only 15% of providers reported feeling uncomfortable providing genetic counseling, whereas over 60% felt comfortable discussing recurrence risk. Fifty percent of participants felt their genetics knowledge was sufficient, whereas 86% expressed interested in furthering their education. Previous work has shown the medical genetics training of health care professionals in LMICs is inadequate in the majority of studies performed to date.^16^ In our sample, however, it appears that providers feel comfortable providing genetic counseling; therefore, initial steps toward educating providers should potentially focus on noncounseling-related topics. An initial topic of focus could be supporting providers in interpreting genetic test reports in the context of a patient’s clinical history—especially because, even without an in-country genetic testing laboratory, many are sending tests abroad. Internal Medicine providers most frequently reported collecting a family history, compared with Pathologists and Surgical Specialists and also felt the most comfortable doing this compared with those same specialists. This information aligns with expected clinical practices of these specialties and suggests starting clinical services in Internal Medicine departments could be a good strategy considering those providers may feel the most comfortable with medical genetics education and training. Alternatively, surgeons and pathologists may be the most in need of the education suggesting their department are a better place to start. Future planning effort in Nepal should consider this experience and need when making decisions regarding medical genetics services in the country.

Despite providing useful information regarding the medical genetics needs of Nepali health care providers, this study does not provide the full picture of what is needed in the country. As mentioned, this convenience sample was primarily based off providers in the Kathmandu Valley and therefore does not fully represent the full spectrum of provider experiences in Nepal. Providers caring for Tharu patients in the Terai region are likely seeing a much higher proportions of recognizable genetic conditions given that sickle cell disease is more recognizable and more prevalent in those communities compared with more rare disorders, such as nonsyndromic neurodevelopmental conditions or inherited cancer syndromes.^17^ Future work should focus on capturing these providers’ experience either through better targeted survey methodologies or direct interactions at their home institutions. We also were unable to compare the differences between respondents and nonrespondents but did note a high percentage of Pathologist participants in this survey. This may be due to author P.G.’s specialty in Pathology with potential participants more likely to respond to her request given their professional familiarity. This potential factor should be considered with future recruitment efforts. Additionally, this was primarily a quantitative study, with limited qualitative data supporting the information reported. A more qualitative exploration of provider preferences and practices, especially surrounding responses with a large percentage of ambivalent or uncertain answers, would provide valuable insights for capacity building efforts. This will be especially important to follow-up on questions raised by the data reported here, such as the types of patients seen daily, for those providers providing very frequent genetic services. We also focused solely on provider perspectives regarding medical genetic services, with no patient representation. Previous work has shown patient interest in medical genetic services across a wide range of specialties, including obstetrics, oncology, and genetic counseling.^18^ Future work should explore patient preferences for medical genetic services because this is a necessary aspect of any medical service implementation project.

In conclusion, the findings show that Nepali providers are regularly seeing patients with genetic conditions. Providers report that the lack of medical genetics education and an in-country diagnostic genetics laboratory are significant barriers to care delivery and should be addressed moving forward. This study adds to the growing body of literature highlighting the importance of medical genetics services in all countries, regardless of income level. Although there are commonalities between countries lacking these services, it is crucial for education and infrastructure efforts to be country and region specific. In countries such as Nepal, with incredible geographic and population diversity, this approach will be particularly important. A 1-size fits all approach will likely further widen and exacerbate disparities in care, especially among vulnerable populations, such as the Tharu.

## Data Availability

The data that support the findings in this article are available on request from the corresponding author, P.G.

## Conflict of Interest

All authors declare no conflicts of interest.
